# The Influence of Metabolic Inhibitors, Antibiotics, and Microgravity on Intact Cell MALDI-TOF Mass Spectra of the Cyanobacterium *Synechococcus* Sp. UPOC S4

**DOI:** 10.3390/molecules26061683

**Published:** 2021-03-17

**Authors:** Marek Šebela, Martin Raus, Vladan Ondřej, Petr Hašler

**Affiliations:** 1 Department of Biochemistry and Centre of the Region Haná for Biotechnological and Agricultural Research, Faculty of Science, Palacký University, Šlechtitelů 27, CZ-783 71 Olomouc, Czech Republic; martin.raus@upol.cz; 2 Department of Botany, Faculty of Science, Palacký University, Šlechtitelů 27, CZ-783 71 Olomouc, Czech Republic; vladan.ondrej@upol.cz

**Keywords:** antibiotic, cyanobacterium, inhibitor, MALDI, metabolism, *Synechococcus*

## Abstract

The aim and novelty of this paper are found in assessing the influence of inhibitors and antibiotics on intact cell MALDI-TOF mass spectra of the cyanobacterium *Synechococcus* sp. UPOC S4 and to check the impact on reliability of identification. Defining the limits of this method is important for its use in biology and applied science. The compounds included inhibitors of respiration, glycolysis, citrate cycle, and proteosynthesis. They were used at 1–10 μM concentrations and different periods of up to 3 weeks. Cells were also grown without inhibitors in a microgravity because of expected strong effects. Mass spectra were evaluated using controls and interpreted in terms of differential peaks and their assignment to protein sequences by mass. Antibiotics, azide, and bromopyruvate had the greatest impact. The spectral patterns were markedly altered after a prolonged incubation at higher concentrations, which precluded identification in the database of reference spectra. The incubation in microgravity showed a similar effect. These differences were evident in dendrograms constructed from the spectral data. Enzyme inhibitors affected the spectra to a smaller extent. This study shows that only a long-term presence of antibiotics and strong metabolic inhibitors in the medium at 10^−5^ M concentrations hinders the correct identification of cyanobacteria by matrix-assisted laser desorption/ionization time-of-flight mass spectrometry (MALDI-TOF).

## 1. Introduction

Cyanobacteria represent a phylum of photosynthesizing bacteria. The small size of cells, overlapping features, and complicated life cycles are the traditional base for their classification. However, the whole taxonomic system has gradually been revised with the introduction of phylogenetic analyses based on molecular sequencing data, which are commonly obtained from specific DNA regions such as 16S rRNA gene and the internal transcribed spacer [1].

Numerous papers have appeared describing the applicability of intact cell matrix-assisted laser desorption/ionization time-of-flight mass spectrometry (IC MALDI-TOF MS) for identification and typing of bacteria, yeasts, and fungi [2,3]. In principle, a sufficient number of cells is picked up from the culture (>10^5^ cells), transferred to the target plate, and overlaid with a matrix solution for co-crystallization. Optionally, especially for pathogenic microorganisms, microbial cells are inactivated and extracted prior to the preparation of MALDI probe. The analysis of cellular pathogens is possible also from positive blood cultures or body fluids [4]. The pattern of molecular masses in the profile MALDI spectrum (usually signals of ribosomal and other abundant proteins are predominantly represented) is finally compared with a reference spectral database to assign the result [2].

This strategy has revolutionized the process of pathogen identification in clinical microbiology laboratories because it is fast, efficient, and inexpensive [5]. There is a general agreement that the results based on IC MALDI-TOF MS are highly reliable, which stems from its reproducibility and accuracy. The identification rates at the genus as well as species levels in experimental studies using culture plates may largely exceed 90% [5,6]. Values over 90% have also been reported for detecting microorganisms from blood cultures or urine. High-confidence identifications from clinical specimens allow clinicians to decide responsibly about the subsequent treatment of infections by the selection of suitable antibiotics [5]. Difficulties may occur when closely related species show similar MALDI-TOF profile spectra and cannot be distinguished in this way [7]. Additionally, data inconsistencies for the same type of cells may appear due to different growth conditions, which can profoundly affect the microbial physiology and protein expression profile [8].

Previous IC MALDI-TOF mass spectra of cyanobacteria reported the presence of signals representing abundant proteins from the 30S and 50S ribosomal subunits, which could easily be assigned based on the molecular mass values [9]. We have recently developed a procedure based on IC MALDI-TOF MS with the optimized FASA matrix system (ferulic and sinapinic acids), which functioned well for the characterization of taxonomic relationships of various *Chroococcidiopsis* isolates including *Ch. cubana* and *Ch. thermalis*. Protein extracts were made by treating cyanobacterial cells with the acidic matrix solvent to identify proteins for assigning the characteristic peaks in IC MALDI-TOF profiles: photosystem components, phycobilisome proteins, porins, electron transport proteins, nitrogen metabolism and nucleic acid binding proteins, cytochromes plus other enzymes, and uncharacterized proteins [10].

To the best of our knowledge, no reports about the influence of metabolic changes on characteristic MALDI spectral profiles of cyanobacteria have been published so far. On the other hand, many comparative proteomics studies appeared describing the response to various environmental stress factors such as UV light irradiation [11], extreme temperature [12,13], increased salinity [14,15], extreme pH [16,17], and heavy metals [18]. The cellular abiotic stress adaptation strategies of cyanobacteria uncovered by many previous proteomics analyses have thoroughly been reviewed in a recent paper [19]. Temperature stress influences membrane fluidity and, for example, two-component signal transduction proteins. DNA repair proteins are upregulated and phycobiliproteins downregulated under low temperature. Heat shock proteins and other molecular chaperones are upregulated during high temperature, acid pH, salt, and UV stress. Heavy metals, UV light doses, and oxidative stress severely impair photosynthesis and carbon metabolism. Membrane ion transporters are upregulated under stress conditions [19].

Cyanobacteria have important ecological roles in the global oxygen production and carbon flux as well as soil fertilization due to nitrogen fixation [20]. Non-toxic cyanobacteria are sources of food for many organisms for example in aquatic ecosystems [21]. Spirulina (*Arthrospira*) is available as a food supplement for human diet with a high nutrition value due to its protein and vitamin content [20]. Toxic cyanobacteria have increasingly been studied for their roles in freshwater harmful algal blooms [22,23]. The unique characteristics including toxicity are species-specific and can be influenced by ecological conditions. Thus, fast and reliable identification of cyanobacteria on the species and even lower (i.e., ecotype) levels has a big importance.

In this work, we focused on the influence of metabolic inhibitors and antibiotics at micromolar concentrations on MALDI-TOF mass spectrometric profiles of the cyanobacterium *Synechococcus* sp. UPOC S4 growing in a liquid culture. Bioinfomatics tools were applied to recognize and evaluate the resulting changes and their impact on reliability of sample identification. Defining the limits of IC MALDI-TOF MS of cyanobacteria is important for its use in biology and applied science. Only a long-term presence of antibiotics and strong metabolic inhibitors (e.g., azide) in the medium (3 weeks) hinders the correct identification of cyanobacteria by MALDI-TOF measurements with intact cells. Additionally, the cyanobacterium was cultured without inhibitors under microgravity conditions with expectable changes in the metabolism. Large differences in the mass spectra occurred only after 9 days of microgravity.

## 2. Results

### 2.1. Research Motivation and Implementation

**Figure 1:**
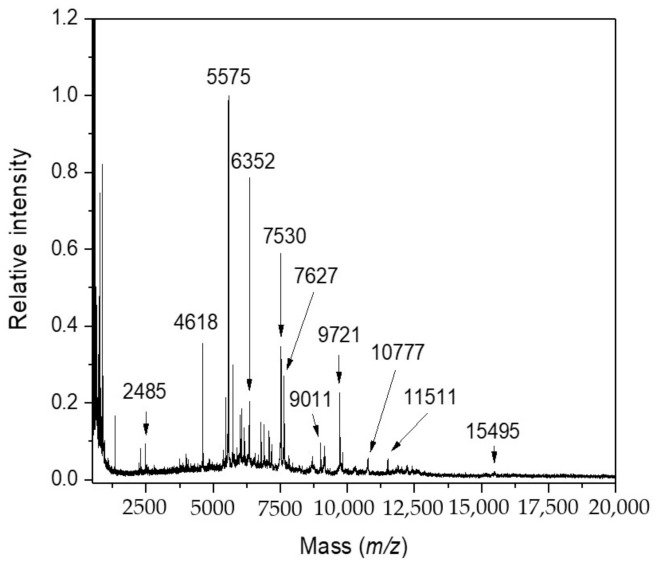
IC MALDI-TOF MS of untreated *Synechococcus* sp. UPOC S4. The spectrum was acquired on Microflex LRF20 MALDI-TOF instrument using the dried-droplet sample preparation technique and FASA (ferulic acid-sinapic acid) binary matrix [10].

*Synechococcus* sp. UPOC S4 cells were cultivated in the presence of metabolic inhibitors and antibiotics to evaluate their impact on the reliability of species identification by IC MALDI-TOF MS. The idea of using *Synechococcus* sp. UPOC S4 as an experimental model system came from our previous research work [10]. This cyanobacterium has been shown to provide intact cell MALDI-TOF mass spectra with many diagnostic peaks, it is easy cultivable, and its proteome sequence is available in the UniProtKB database. The set of compounds included inhibitors of central metabolic pathways such as glycolysis (3-bromopyruvic acid, 2-deoxyglucose) [24,25,26,27,28,29], citric acid cycle (2-fluoroacetic acid, malonic acid) [30,31,32], respiratory chain (antimycin A, sodium azide) [33,34,35,36,37], and antibiotics targeting protein synthesis (chloramphenicol, streptomycin) [38,39,40]. We selected long-known and potent inhibitors, which can be considered archetypical. It was expected that these compounds could largely affect the overall metabolism and cellular status due to their interference with key biochemical processes. In addition, the cultivation of chemically untreated cells was carried out under microgravity conditions. The Random Positioning Machine used in this experiment consists of two frames (an inner and outer frame), which rotate independently from each other in a random direction. A sample placed in the middle of the frames is thus subjected to a weightlessness as the gravity vector is averaged to zero over time [41,42]. Such experiments were chosen because of the expected strong effect on the cells, which could set up limits for the applicability of MALDI-based identification of cyanobacteria. The concentration range used for the chemicals (1–10 μM) was based on previous reports on a growth inhibition of cyanobacteria or green algae by antibiotics and antimetabolites (see the discussion). Differences in peptide/protein MALDI-TOF mass spectrometric profiles were observed as a consequence of the treatment and evaluated by comparing experimental data with the respective controls. Figure 1 shows a standard IC MALDI-TOF mass spectrum of untreated *Synechococcus* sp. UPOC S4 cells. Typically, the Biotyper software assigned around 100 species-characteristic peaks. This one, and other controls, provided identification score values of ≥2.0 (green color; reliable identification at the species level) when analyzed against the library of reference spectra of cyanobacteria, which has continuously been extended since our previous project [10].

**Figure 2:**
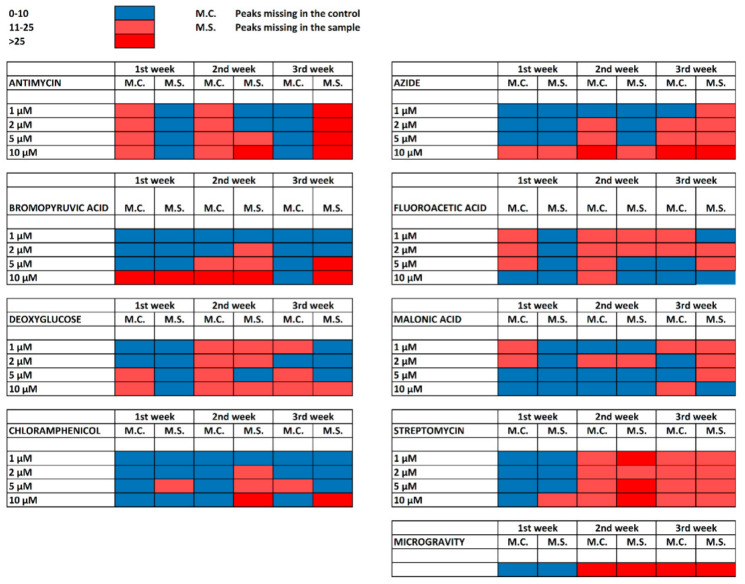
Heat maps with peak differences in the intact cell matrix-assisted laser desorption/ionization time-of-flight mass spectrometry (IC MALDI-TOF) mass spectra for all treatments and incubation periods.

Figure 2 shows a summary of results in the form of heat maps illustrating the level of peak differences in the mass spectra for all treatments and incubation periods. The differential peaks were identified by combining MALDI Biotyper Compass Explorer, pFilter, and mMass software (the latter for a visual inspection of peaks). A list of assigned proteins with known annotations in the UniProtKB database or annotations obtained using a conserved domain search is provided in Supplementary File S1. The following chapters of the results section provide summarizing information to the effect of each analyzed compound as well as microgravity conditions.

### 2.2. Cultivation with Antimycin

The presence of antimycin A in the cultivation medium largely influenced IC MALDI-TOF mass spectra of *Synechococcus* sp. UPOC S4 already after 1 week at 1 and 2 μM concentrations. Around 20 peaks were newly found at 5 and 10 μM concentrations. Uncharacterized proteins for which no conserved domain could be searched represented a majority of hits. Yet, some interesting proteins with available database annotations could be assigned, e.g., *m/z* 3443.74 to PSBT_SYNR3 photosystem II reaction center protein T, *m/z* 4361.43 to Q05XI0_9SYNE cytochrome b6-f complex subunit 7, *m/z* 6370.62 to Q05WR3_9SYNE possible high light inducible protein, *m/z* 8677.30 to K9RU07_SYNP3 ferredoxin, *m/z* 9067.28 to Q0IBQ1_SYNS3 Nif11 (nitrogen fixation) domain-containing protein, *m/z* 9128.53 to NDHO_SYNPW NAD(P)H-quinone oxidoreductase subunit O, and *m/z* 12576.10 to K9SR95_9SYNE photosystem II reaction center Psb28 protein.

**Figure 3:**
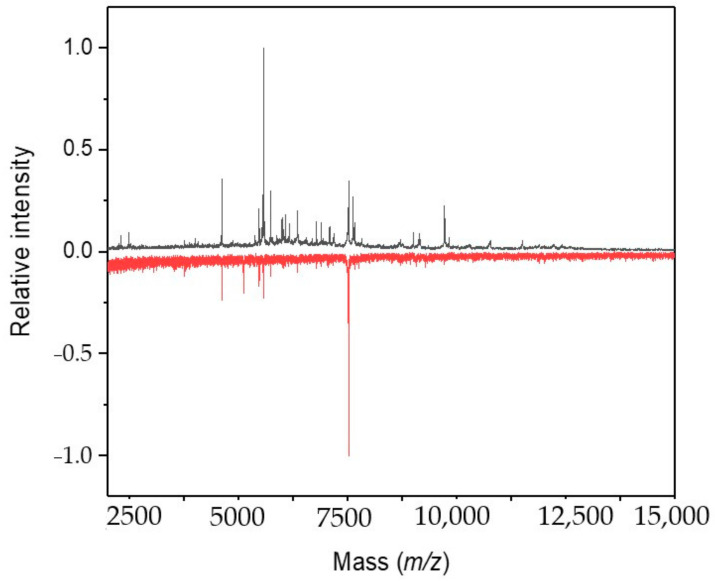
IC MALDI-TOF MS of *Synechococcus* sp. UPOC S4 grown in the presence of antimycin A. The mass spectra refer to the control and sample (cultivated in the presence of 10 µM antimycin A for 3 weeks) and were acquired on Microflex LRF20 MALDI-TOF instrument using the dried-droplet sample preparation technique with FASA binary matrix; top spectrum in black—control; bottom spectrum in red—treated cells (intensities are multiplied by −1 for clarity).

A strong impact of antimycin A was observed after 3 weeks. Whereas only three peaks were newly registered at 1 and 2 μM concentrations, 31 signals completely disappeared from the spectrum when compared to the control. These were assigned, except for uncharacterized and a few other annotated proteins, as follows: *m/z* 4429.12 to A0A1J0PAG3_9SYNE photosystem II reaction center protein I, *m/z* 4858.50 to Q3AY61_SYNS9 possible high light inducible protein, *m/z* 6680.04 to K9SUE7_9SYNE copper chaperone, *m/z* 7084.66 to B1XKL5_SYNP2 chaperone protein domain protein, *m/z* 7193.64 K9RSN2_SYNP3 photosystem II reaction center protein K, and *m/z* 9142.47 to NDHL_SYNPW NAD(P)H-quinone oxidoreductase subunit L. The increased concentrations of 5 and 10 μM resulted in a disappearance of most signals (Figure 3).

### 2.3. Cultivation with Sodium Azide

**Figure 4:**
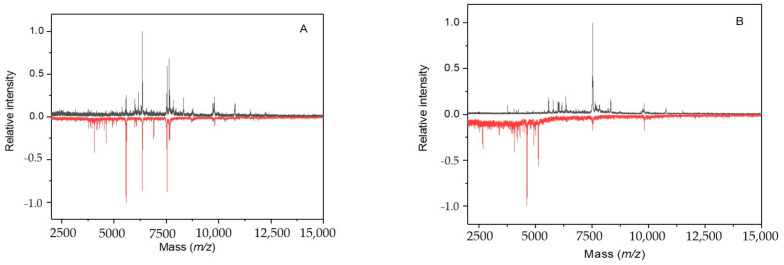
IC MALDI-TOF MS of *Synechococcus* sp. UPOC S4 grown in the presence of sodium azide. The spectra were acquired on Microflex LRF20 MALDI-TOF instrument using the dried-droplet sample preparation technique with FASA binary matrix. Subfigures (**A**,**B**) show results after cultivation with 10 µM azide for 2 and 3 weeks, respectively: top spectrum in black—control; bottom spectrum in red—treated cells (intensities are multiplied by −1 for clarity).

Obvious differences in the IC MALDI-TOF mass spectra of *Synechococcus* sp. UPOC S4 were observed after 2 weeks of incubation, particularly at 5 and 10 µM sodium azide (Figure 4A). Peaks, which newly appeared compared to the control, were correlated mostly with uncharacterized proteins, photosynthesis proteins (e.g., *m/z* 4112.87-A0A164BUR5_9SYNE, *m/z* 4297.12-A0A164B792_9SYNE, *m/z* 5224.27 with PSBK_SYNPW, and *m/z* 7047.75-A0A2P7EC83_9SYNE), and redox enzymes (*m/z* 6454.65-A5GI63_SYNPW). Peaks from the control missing in the sample spectrum were assigned e.g., to proteins from photosynthetic complexes (*m/z* 6935.76 to A0A0G8ARK5_9SYNE, *m/z* 12403.77 to B4WSH1_SYNS7, and others), translocase (*m/z* 8724.39 to A0A2P7EFI8_9SYNE), high light inducible protein (*m/z* 8764.02 to Q3AYN7_SYNS9), and antitoxin (*m/z* 8764.02 to Q05TX2_9SYNE).

Prolonged treatments for 3 weeks again provided a marked change in the mass spectrum, particularly when 10 µM azide was applied (Figure 4B). The concentration of 10 µM induced a new appearance of 33 peaks assigned to uncharacterized proteins and frequently also to components of photosynthetic complexes (e.g., *m/z* 3594.45 to PETN_SYNPW, *m/z* 3812.22 to PSBM_SYNR3; five other peaks between *m/z* 4200 and 5000 matched by their masses to photosystem I and II subunits). Control peaks (44) missing in the sample spectrum were attributed to uncharacterized proteins, membrane proteins and enzymes (e.g., *m/z* 5272.37 to A5GN15_SYNPW, *m/z* 6408.12 to K9SVD1_9SYNE) and redox enzymes (e.g., *m/z* 7963.73 to NDHO_SYNJB, *m/z* 9741.52 to A4CXC4_SYNPV). Other correlated peaks included, e.g., *m/z* 6788.29 (A0A4V1DI31_9SYNE, high light inducible protein), *m/z* 7663.31 (K9RVM2_SYNP3, NAD(P)H dehydrogenase subunit S), and *m/z* 7963.73 (A0A1J0PC15_9SYNE, thiamine biosynthesis protein ThiS).

### 2.4. Cultivation with 3-Bromopyruvic Acid

The growth of *Synechoccocus* sp. UPOC S4 cells in the presence of 1–5 µM 3-bromopyruvic acid (BrPA) for 1 week yielded virtually unchanged IC MALDI-TOF mass spectra. More differences were registered after 2 weeks. Newly appeared peaks in the sample spectrum (almost 30 at 10 μM BrPA after 1 week, which disappeared after 2 weeks) were attributed mainly to uncharacterized proteins, components of photosynthetic complexes (*m/z* 3517.27 to PETN_SYNR3, *m/z* 7307.50 to A0A4P7ZUJ9_9SYNE), electron carriers (*m/z* 8025.83 to K9RWY0_SYNP3 ferredoxin), some enzymes (*m/z* 7130.37 to A3Z2C8_9SYNE), binding proteins (*m/z* 9037.00 to A5GUA2_SYNR3, *m/z* 9802.61 to Q3AZD0_SYNS9), and others. Peaks missing in the sample spectrum were assigned to numerous uncharacterized proteins, proteins from photosynthetic complexes (e.g., *m/z* 3765.29 to K9SW16_9SYNE, *m/z* 4432.62 to PSAJ_SYNR3), membrane proteins (e.g., *m/z* 6070.47 B4WRS1_SYNS7, *m/z* 7517.90 to A5GRA1_SYNR3), redox enzymes (e.g., *m/z* 6426.47 to A0A164CQ37_9SYNE, *m/z* 7664.05 to K9RVM2_SYNP3), other enzymes (e.g., *m/z* 6070.47 to B4WRS1_SYNS7, *m/z* 8206.35 to Q3AUQ7_SYNS9), and protein factors and regulators (e.g., *m/z* 9721.41 to Q3AZ30_SYNS9, *m/z* 9738.37 to A0A4P7ZUR7_9SYNE). The treatment with 1 and 2 μM BrPA for 3 weeks resulted only in a few differential peaks in IC MALDI-TOF MS peptide/protein profile. After the treatment with 5 and 10 μM BrPA, many signals from the control spectrum were missing in the sample spectra, in the latter case almost all.

### 2.5. Cultivation with 2-Deoxyglucose

One week of incubation with 1 and 2 μM 2-deoxy-D-glucose (DG) provided less than 10 differential peaks correlated with uncharacterized proteins. Higher concentrations of DG resulted in almost 20 peaks missing in the control assigned mostly to uncharacterized proteins or photosynthesis-related proteins. Almost all differences, however, were bound to peaks with low intensities. They did not influence the spectral pattern too much, which allowed clear identifications at the species level. The number of peaks, which newly appeared in the sample after 2 weeks of incubation, was 10–25. Again, attempts to assign them to *Synechococcus* sequences resulted in uncharacterized proteins, photosystems components (*m/z* 3812.30 to PSBM_SYNR3, *m/z* 6664.24 to PSBZ_SYNJB), high light-induced proteins (*m/z* 5803.10 to Q3AZA1_SYNS9, *m/z* 7922.45 to A3YYG0_9SYNE), electron transfer proteins (*m/z* 5982.24 to A0A4P7ZY82_9SYNE, *m/z* 7944.18 to A0A1J0PCN5_9SYNE), and some others. Similarly, around 10 peaks missing in the sample spectra could be attributed to uncharacterized proteins and then also to photosynthesis-related proteins such as ferredoxin (*m/z* 11844.24 to Q3AZL8_SYNS9) or thioredoxin (*m/z* 11736.71 to A4CRP3_SYNPV) at 5 and 10 μM DG. Similar numbers of differential peaks and assignments to photosystems components, high light-induced proteins and electron carrier proteins were observed after 3 weeks of incubation.

### 2.6. Cultivation with 2-Fluoroacetic and Malonic Acids

**Figure 5:**
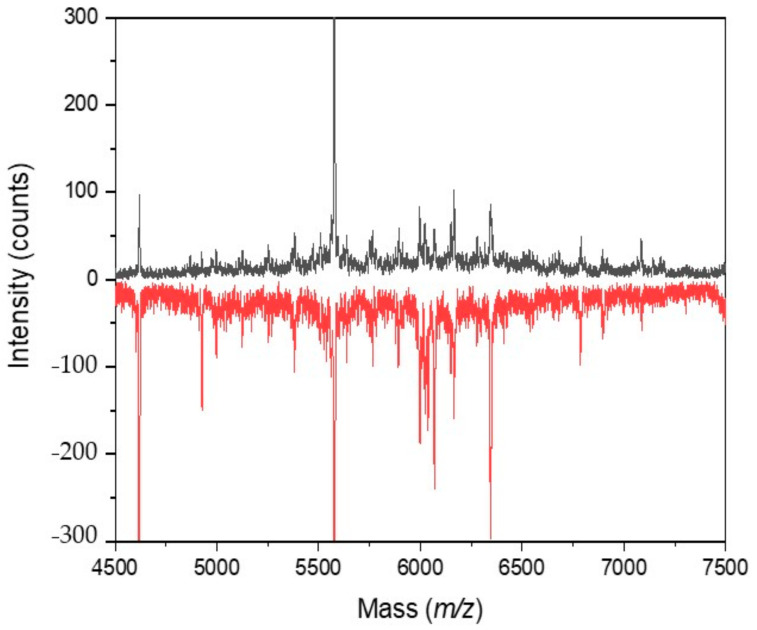
IC MALDI-TOF MS of *Synechococcus* sp. UPOC S4 grown in the presence of FAA. The figure shows a close-up view of the *m/z* region 4500–7500 and refers to the control and sample cultivated in the presence of 2 µM FAA for 2 weeks. The spectra were acquired on Microflex LRF20 MALDI-TOF instrument using the dried-droplet sample preparation technique with FASA binary matrix; top spectrum in black—control; bottom spectrum in red—treated cells (intensities are multiplied by −1 for clarity).

The overall appearance of IC MALDI-TOF mass spectra of *Synechococcus* sp. UPOC S4 cultivated in the presence of 1–10 µM 2-fluoroacetic acid (FAA) for 1–3 weeks changed moderately, which did not influence spectral-based identifications at the species level (Figure 5). Peaks that newly appeared after 3 weeks of incubation (up to 20) could be attributed to uncharacterized proteins, photosynthesis proteins (e.g., *m/z* 3874.73 to K9RVZ0_SYNP3, *m/z* 4020.79 to A0A4P7ZWX4_9SYNE, and *m/z* 6705.21 to Q0IE04_SYNS3), high light inducible proteins (*m/z* 6993.79 to A5GK98_SYNPW), and some other proteins including *m/z* 8714.28 assigned to A5GPD7_SYNPW protein translocase subunit SecE and *m/z* 7645.26 to A4CYF4_SYNPV translocase. After 3 weeks at 1–10 μM FAA, the sample spectra did not contain 10–20 peaks registered in the control. Apart from uncharacterized proteins, these peaks were assigned, e.g., to some photosynthesis proteins, molybdenum-pterin binding domain protein (*m/z* 7305.72 to Q2JP93_SYNJB), and antitoxin (*m/z* 9013.85 to Q05TX6_9SYNE).

Similar to the results with FAA, the presence of malonic acid (MA) in the cultivation medium for a period of up to 3 weeks did not change IC MALDI-TOF mass spectra of *Synechoccocus* sp. UPOC S4 enough to preclude identifications at the species level. Differential peaks were typically those with a low intensity. Two and three weeks of incubation at 1–10 μM MA were reflected in 8–16 differential peaks compared to the control. Sequence-based assignments resulted in uncharacterized proteins, photosynthesis-related proteins such as chlorophyll A-B binding proteins (*m/z* 6115.37 to K9SXK0_9SYNE, *m/z* 6364.76 to K9RMW4_SYNP3, *m/z* 7557.35 to K9RTG0_SYNP3) and components of photosynthetic complexes (e.g., *m/z* 5199.17 to PSBK_SYNR3, *m/z* 7560.45 to PSBH_SYNJB). Peaks missing in the sample were apart from uncharacterized proteins attributed to photosynthesis (e.g., *m/z* 9106.97 to PSBE_SYNS3, *m/z* 7686.81 to A0A164BQV3_9SYNE), nutrition-related proteins (e.g., *m/z* 8313.43 to A0A164BYD7_9SYNE, *m/z* 12250.37 to K9SS56_9SYNE), antitoxins, and, for example, cell division protein SepF (*m/z* 10164.11 to A0A4P7ZXV3_9SYNE).

### 2.7. Cultivation with Chloramphenicol

The effect of chloramphenicol on the resulting IC MALDI-TOF mass spectra of *Synechococcus* sp. UPOC S4 was negligible after 1 week of cultivation. More differences were registered after 2 weeks, particularly as peaks missing in the IC MALDI-TOF mass spectra of treated samples. The applied concentrations of 1, 2, and 5 μM were accompanied by 5–16 differential peaks. The missing peaks were attributed typically to uncharacterized proteins, components of photosynthetic complexes (*m/z* 3764.80 to K9SW16_9SYNE), light inducible proteins (*m/z* 4859.77 to Q3AY61_SYNS9, *m/z* 7176.96 to A5GNP5_SYNPW), redox enzymes (*m/z* 6920.06 to B4WJ98_SYNS7, *m/z* 8710.03 to A0A163YG82_9SYNE), binding and transport proteins (e.g., *m/z* 6095.00 to A4CU00_SYNPV, *m/z* 8315.07 to A0A4P7ZV37_9SYNE), and regulators (*m/z* 5710.98 to B4WGD5_SYNS7, *m/z* 9830.71 to A0A4P7ZS91_9SYNE). A majority of peaks from the control were missing in the sample spectrum in the presence of 10 μM chloramphenicol for 2 weeks. The IC MALDI-TOF mass spectrum of *Synechococcus* sp. UPOC S4 cells markedly changed after 3 weeks of cultivation in the presence of 10 μM chloramphenicol, whereas the differences at lower concentrations were weaker. The number of peaks that newly appeared for the sample compared to the control spectrum was around 10 at 1–5 μM concentration. On the contrary, only a few peaks were missing in the sample spectrum. The differential peaks could be assigned only to uncharacterized proteins. Almost all signals were missing compared to the control at 10 μM chloramphenicol.

### 2.8. Cultivation with Streptomycin

The differences compared to the control were minute after 1 week of treatment, especially at low streptomycin concentrations of 1 and 2 μM. More changes were found after 2 weeks. Newly appeared peaks (up to 20) were attributed mainly to uncharacterized proteins, proteins from photosynthetic complexes (e.g., *m/z* 3765.63 to K9SW16_9SYNE, *m/z* 8711.30 to K9SS39_9SYNE), redox enzymes (e.g., *m/z* 5170.29 to Q05UQ6_9SYNE, *m/z* 12024.07 to Q3B072_SYNS9), other enzymes (*m/z* 12578.41 to A0A4P7ZVA0_9SYNE), transcription regulators (*m/z* 7532.70 to A0A4P7ZV92_9SYNE), and some other proteins. The peaks missing in the sample spectra (up to 36) were assigned typically to uncharacterized proteins, proteins from photosynthetic complexes (*m/z* 4505.66 to PSBL_SYNS3, *m/z* 5842.06 to B4WII5_SYNS7), redox enzymes (e.g., *m/z* 6166.07 to A4CU28_SYNPV, *m/z* 7963.08 NDHO_SYNJB), other enzymes (e.g., *m/z* 5983.44 Q05TX4_9SYNE, *m/z* 6408.23 to K9SVD1_9SYNE-Sec-independent protein translocase protein TatA), antitoxins (*m/z* 7948.06 to B4WFM6_SYNS7, *m/z* 9012.75 to Q05TX6_9SYNE), protein related to nutrition and transport (*m/z* 9829.88 to B1XMV8_SYNP2), and regulators (*m/z* 14172.83 to A5GUG5_SYNR3). The presence of 1–10 μM streptomycin in the medium for 3 weeks was reflected in a new appearance of 10–20 peaks. Numerous signals were missing in the sample spectra compared to the control.

### 2.9. Cultivation under Microgravity Conditions

**Figure 6:**
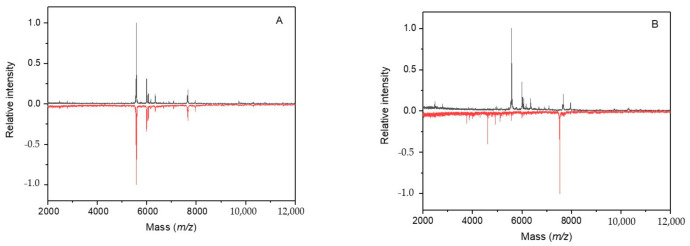
IC MALDI-TOF MS of *Synechococcus* sp. UPOC S4 grown in microgravity conditions. The spectra were acquired on Microflex LRF20 MALDI-TOF instrument using the dried-droplet sample preparation technique with FASA binary matrix. Subfigures (**A**,**B**) show results after cultivation for 6 and 17 days, respectively: top spectrum in black—control; bottom spectrum in red—treated cells (intensities are multiplied by −1 for clarity).

The growth of *Synechococcus* sp. UPOC S4 cells under simulated microgravity yielded gradual changes in the IC MALDI-TOF mass spectrum. Obvious alterations in the profile spectra were registered after 9 days of cultivation in the Random Positioning Machine and were striking after more than 2 weeks (Figure 6). Then, the number of peaks that newly appeared in the sample spectrum compared to the control reached a value of 35. Based on the measured *m/z* values, these differential peaks were mostly correlated with uncharacterized proteins plus, e.g., *m/z* 4043.25 with PSBJ_SYNJB photosystem II reaction center protein J, *m/z* 4989.10 and 5120.49 with high light inducible proteins (Q05RZ3_9SYNE and A0A1J0PBS6_9SYNE, respectively), and *m/z* 9146.22 with Q3AVY9_SYNS9 glutaredoxin. A higher number of 44 peaks from the control were found missing in the sample spectrum. They were typically attributed to uncharacterized proteins, proteins from photosynthetic complexes (e.g., *m/z* 4977.06 to B4WKQ1_SYNS7, *m/z* 4994.44 to A3YTM9_9SYNE), light inducible proteins (*m/z* 4962.98 to A3YVC3_9SYNE, *m/z* 5099.57 to A3YW30_9SYNE), redox enzymes and other redox proteins (e.g., *m/z* 6427.62 to Q05UF0_9SYNE, *m/z* 10781.42 to A4CVU0_SYNPV), antitoxins (*m/z* 7087.99 to A3Z0W8_9SYNE, *m/z* 8330.00 to A3YV18_9SYNE), and regulators (*m/z* 8578.39 to Q3AUX7_SYNS9).

## 3. Discussion

The acquisition of IC MALDI-TOF mass spectra of cyanobacteria has been optimized in our laboratory using binary FASA matrix [10]. There is a database of around 120 reference spectra currently available, including *Synechococcus* isolates, which can be searched for species identification.

**Figure 7:**
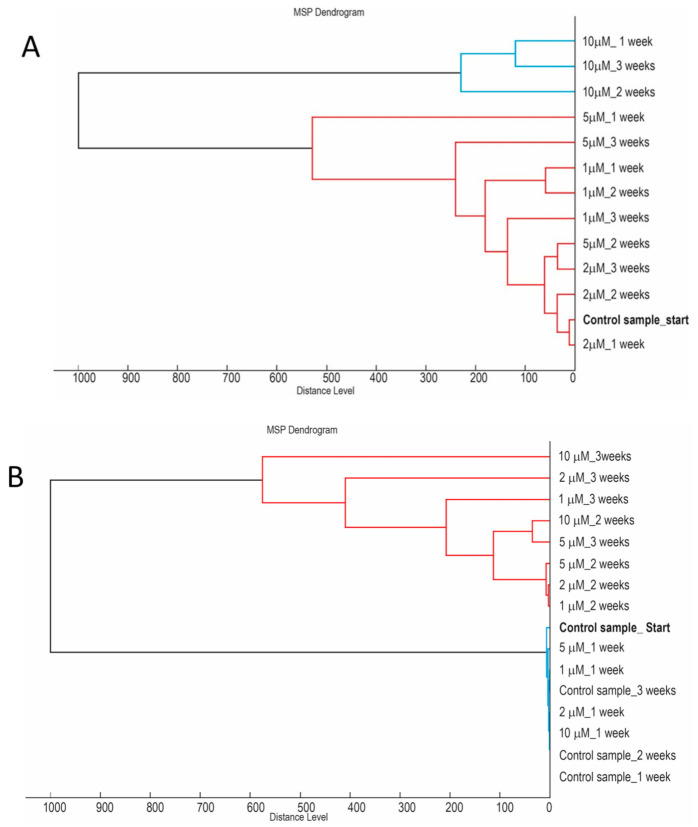
Hierarchical trees of *Synechococcus* sp. UPOC S4 cells based on IC MALDI-TOF mass spectra. Multiple spectra were processed for samples cultivated in the presence of sodium azide (**A**) and streptomycin (**B**) in MALDI Biotyper Compass Explorer 4.1 (Bruker Daltonik) to generate main spectral projections (MSPs). A library of the MSPs was then used for tree construction.

This study was aimed at elucidating how the presence of chemical compounds, which strongly interfere with biological and biochemical processes in the cell, influences the reliability of MALDI-based identification of the cyanobacterium *Synechococcus sp*. UPOC S4 at the species level. This was stimulated by warnings and doubts appearing in the related literature that the metabolic status of analyzed microbial cells can largely influence their spectral profiles and make them divergent from reference spectra in databases. The present IC MALDI-TOF MS spectra of *Synechococcus sp*. UPOC S4 cells treated by metabolic inhibitors and antibiotics together with the respective controls were evaluated by a hierarchical clustering analysis for discussing differences in the degree of cellular damage. Figure 7 shows dendrograms obtained for the presence of sodium azide and streptomycin in the cultivation medium. As can be seen in Figure 7, panel A, spectra reflecting the highest azide concentration of 10 µM clustered together regardless of the incubation time. A similar kind of clustering was observed for BrPA (Supplementary Figure S1). The spectral changes induced by streptomycin (Figure 7, panel B) were principally different as all incubations at 1–10 µM for 2 and 3 weeks clustered together. This was found to also be rather similar for the other studied antibiotics. Much less harmful compounds as regards the induced spectral changes such as MA provided dendrograms with clusters, which did not correlate completely with the incubation period (Supplementary Figure S1). In the experiment with microgravity cultivation, the IC MALDI-TOF spectra acquired on day 9 and later on clustered outside the others with differences progressing in time within this cluster (Supplementary Figure S1).

The impact of antibiotics on non-target organisms in aquatic ecosystems is studied as these chemicals appear as common contaminants (~0.02–0.025 μg·L^−1^) in waste waters from industry and agriculture [43]. Cyanobacteria have repeatedly been confirmed more sensitive to the toxic effect of antibiotics than green algae, but this is not a fixed rule and it depends on a particular compound [44,45]. Standard toxicity tests are based, for example, on measurements of growth inhibition or inhibition of photosynthetic yield [43,44]. Streptomycin toxicity against the cyanobacterium *Microcystis aeruginosa* and the green alga *Chlorella vulgaris* was previously evaluated by determining half-maximal effective concentration (EC_50_) values after a treatment for 96 h [46]. The determined EC_50_ for *M. aeruginosa* was only 0.29 mg·L^−1^ (~0.5 µM). This concentration level inhibited cell growth, damaged cell membrane, induced antioxidant enzymes, and decreased the content of photosynthetic pigments. Streptomycin also inhibited transcription of photosynthesis-related genes (e.g., those coding for photosystem components), which is consistent with our results. The higher sensitivity to streptomycin compared to *Chlorella* was elucidated in terms of an increased formation of reactive oxygen species [46]. The experimental concentrations of chemicals of 1–10 μM used in this work were chosen with an expectation to have moderate effects in a chronic exposure for weeks. To the best of our search efforts, the relevant literature information is very limited. No previous data on a treatment of *Synechococcus* by 2-deoxyglucose could be found. However, another sugar antimetabolite, 7-deoxy-sedoheptulose, inhibited *Anabaena variabilis* at 50 μM concentration [47]. In *Gloeocapsa*, 1 mM FAA markedly inhibited metabolic pathways [48]. A minimum bactericidal concentration of BrPA of 40 μg mL^−1^ (~0.25 mM) after 2 h of treatment was found for *Staphylococcus aureus* [49]. Sodium azide was found bacteriostatic at 0.01–0.03 % w/v concentrations corresponding to low millimolar values [50]. Although it interferes with a number of biological processes, the most critical for microbial growth seems to be its impact on the protein translocation machinery in the cell membrane as azide inhibits SecA ATPase from the translocase system [51]. An EC_50_ value of 0.35 mg·L^−1^ (5.4 µM) has been reported for azide toxicity towards aquatic microorganisms such as green algae (after 96 h of exposure) [52]. It is highly probable that response to antibiotics, stress, and toxicity is species- and strain-specific. Transport through the external cell wall layer can be affected by its thickness and structure. Mucilaginous envelopes and S-layers, which serve as a protective coat or molecular sieve, were found in members of the genera *Synechococcus*, *Synechocystis*, *Microcystis*, *Gloeocapsa*, and others [53]

Attributing peaks from IC MALDI-TOF mass spectra to *Synechococcus* protein sequences by matching experimental masses was achieved in pFilter according to a previous strategy [54]. The studied compounds do not modify proteins covalently except for the alkylating effect of BrPA on its target enzymes in glycolysis. A mass tolerance window of ±2 Da was used as the spectra were measured with a reasonable accuracy provided by the reflectron mode and external calibration (for average masses) with error values below 50 pm. This way of peak assignment should be taken with caution (for example it does not consider posttranslational modifications) and cannot substitute for a sequencing analysis. In addition, a significant part of the database sequences refers to yet uncharacterized proteins. On the other hand, some of the assigned proteins were previously identified by nLC-ESI-MS/MS in *Synechococcus* sp. UPOC S4 extracts including various photosystem I and II reaction center proteins, high light inducible proteins, cytochrome b559 subunit beta, NAD(P)H-quinone oxidoreductase subunits, phycobilisome 7.8 kDa linker polypeptide, translocase subunits, and others [10]. The cell damage in the presence of toxic chemicals (notably azide and BrPA) or antibiotics increased with time and appeared extensive after 3 weeks of incubation. We suppose that namely photosynthesis, redox reactions, transport, and nutrition supply were impaired and stress responses activated. This is in agreement with the already mentioned study on the impact of streptomycin on *Microcystis*, which indicated increased oxidative stress and reduced transcription of photosynthesis-related genes [46]. High light inducible proteins play a role in non-photochemical energy quenching, stabilizing, and photoprotection of photosystem II as well as in chlorophyll biogenesis [55]. It is then consistent that they were frequently assigned to newly appeared peaks after the chemical treatments. Importantly, the effect of azide was found associated with the Sec translocase system, which has been reported azide-sensitive [51].

Similar to the results of chemical treatment, it seems that the microgravity cultivation again influenced namely photosynthesis and redox metabolism. Moreover, stress responsive mechanisms might be implicated with a high probability, for example, the toxin-antitoxin system as the peaks attributed to antitoxins were frequently missing in spectra of the treated samples. Toxins can inhibit growth, which allows cells to enter a stress-tolerant state, or the cells may even die because of targeting important cellular structures or processes such as membrane integrity, cell wall biosynthesis, replication, and translation [56]. The antitoxin has a neutralizing role towards the effect of toxin. As the toxin is more stable and effective for a longer period, cells need to constantly produce antitoxin in order to survive. A comprehensive review on proteomics of responses to abiotic stress in cyanobacteria has recently been published, which points out the role of proteins. The adaptation mechanisms involve resynthesis of photosystem proteins (proteins related to photosynthesis represent the main category of cyanobacterial proteomes and abiotic stresses are harmful to photosynthesis), and production of antioxidative redox proteins (including thioredoxin and glutaredoxin), molecular chaperones, transporters, transcription regulators, DNA repair proteins, etc. [19].

The largely damaged cells, which are definitely not in a vital state ensuring proper conditions for IC MALDI-based identification, lost the ability to provide a majority of signals observed in the respective control spectra. This was reflected in lowered identification rates. The samples treated by DG, FAA, and MA could be identified in Biotyper at the species level even after the incubations for 3 weeks (with the exception of the highest inhibitor concentration of 10 μM allowing only genus-level identifications). Conversely, antimycin- and streptomycin-treated cells could not be identified after the two weeks of treatment at 1–10 μM, whereas in the case of azide, only the concentrations of 5 and 10 μM showed this harsh effect similarly to BrPA at 10 μM. The weakest effect in this regard among the studied antibiotics was observed for chloramphenicol where only 10 μM concentration was deteriorating (down to genus-level identifications). Finally, microgravity incubation precluded any reliable identification after 9 days. Taken together, we show that only an extensive cell damage of cyanobacteria due to long incubations with toxic chemicals or antibiotics prevents effective MALDI-based identification with intact cells. Common cultivations, especially when photosynthesis is not impaired, should therefore provide reliable data. We consider IC MALDI-TOF MS very useful for a fast, precise, and cheap identification of cyanobacteria in taxonomy or applied science as it remains reliable enough even in situations when the living cells are stressed and challenged by chemicals damaging their metabolism.

## 4. Materials and Methods

### 4.1. Chemicals

Antimycin A, 3-bromopyruvic acid (BrPA), 2-deoxy-D-glucose (DG), ferulic acid, 2-fluoroacetic acid (FAA), malonic acid (MA), sinapinic acid, and sodium azide were of the highest purity available from Sigma-Aldrich Chemie (Steinheim, Germany). Chloramphenicol and streptomycin were from Duchefa Biochemie (Haarlem, the Netherlands). Organic solvents and water (LC-MS grade) were from Merck (Darmstadt, Germany). All other chemicals were of analytical purity grade.

### 4.2. Cultivation of Untreated Cyanobacterial Cells

The experimental strain *Synechococcus* sp. UPOC S4 represents a coccal cyanobacterium from the order Synechococcales. It was isolated in 2006 as an endozoic living inside *Paramecium bursaria* from a small pool near Loštice, Czech Republic. The strain was cultivated in Zehnder medium [57] under the following conditions: light regime of 14 h light and 10 h dark; light irradiation of 20 μmol.m^−2^.s^−1^; temperature of 22 °C. *Synechococcus* cells grew in a liquid culture.

### 4.3. Cultivation of Treated Cyanobacterial Cells

A sufficient amount of biomass was pre-cultivated after inoculation in 1 l Erlenmeyer flasks for 14 days. Subsequently, 10 mL aliquot samples were separated into sterile test tubes. Four samples were supplemented with each evaluated chemical compound or antibiotic at different concentrations of 1, 2, 5, and 10 μM. The fifth sample served as an untreated control. The samples (2 replicates) were incubated up to 21 days under the same laboratory conditions as described above and analyzed in 1-week intervals. The pre-cultivated suspension contained 4.2 × 10^5^ cells per mL with an optical density value OD_750_ of 1.28. To estimate the biological effect of the analyzed compounds, we tested azide as an example of well-known strong inhibitor, initially at 1 and 10 mM, as millimolar concentrations have been described as bacteriostatic [50]. After one week, the OD_750_ value for the control culture increased by 0.10, while in the presence of 1 mM azide it decreased by 0.16 and for 10 mM even by 0.23. It stagnated at around 1.3 for 10 µM azide indicating a much weaker effect. For IC MALDI-TOF MS, the cells were always freshly collected by centrifugation [10] and suspended in MS-quality water to achieve >5 × 10^7^ cells per mL. *Synechococcus* sp. UPOC S4 cells were also grown in a liquid culture under low gravity conditions simulated by a Random Positioning Machine (RPM; Dutch Space, Leiden, the Netherlands). The instrument contains an inner and outer frame with a platform to place samples in the middle. The frames rotate independently of each other. Test tubes with 10 mL samples were fixed in the center of the inner frame. The maximum and minimum angular velocity of rotation was 50° s^−1^ and 40° s^−1^. The samples were under a simulated microgravity of 0.1× *g* at 22 °C. The controls were obtained as above without using microgravity. The period of the experiment was also 3 weeks. However, as we anticipated that low gravity conditions would largely influence the physiology and metabolism of the cyanobacterium, MALDI measurements were performed at shorter time intervals.

### 4.4. Mass Spectrometry

IC MALDI-TOF MS was performed on a Microflex LRF20 mass spectrometer (Bruker Daltonik, Bremen, Germany) equipped with a nitrogen laser (λ_max_: 337 nm, pulse repetition rate: 60 Hz). Mass spectra were acquired in the reflectron positive ion mode using an acceleration voltage (IS1) of 19.0 kV, extraction voltage (IS2) of 15.5 kV, lens voltage of 9.0 kV, reflectron voltage of 20.0 kV, detector voltage of 1668 V, and pulsed ion extraction delay time of 500 ns. The mass region for examination ranged from *m/z* 1000 to *m/z* 25,000. Protein Calibration Standard I (Bruker Daltonik), covering average [M+H]^+^ masses between 5734.5 and 16952.3 Da, was applied for an external calibration. The FASA matrix system consisted of ferulic acid (FA; 5 mg·mL^−1^) and sinapinic acids (SA; 15 mg·mL^−1^) dissolved in a mixture of acetonitrile and 2.5 % (*v*/*v*) trifluoroacetic acid, 7:3, *v*/*v*. The analyzed cell suspension (1 μL) was applied onto the target plate (MSP BigAnchor 96 BC microScout Target; Bruker Daltonik), mixed with the same volume of matrix solution and left to dry for crystallization [10]. The acquisition and evaluation software were flexControl 3.4 and flexAnalysis 3.4 (all by Bruker Daltonik), respectively. IC MALDI-TOF mass spectra were accumulated from 1000 laser shots and averaged from different positions at the sample spots (a random target movement was applied).

### 4.5. Data Processing

MALDI-TOF mass spectra of intact cells cultivated in the presence of inhibitors or antibiotics as well as those from the microgravity cultivation were processed by making comparisons with the respective controls. Specific peaks were identified in the acquired spectra either using MALDI Biotyper Compass Explorer 4.1 software (Bruker Daltonik) or Biospean [58] available at https://software.cr-hana.upol.cz/biospean/. The input data for Biospean were in TXT format as exported from the flexAnalysis 3.4 spectra evaluation software (Bruker Daltonik). Main spectra projections (MSPs) were constructed in MALDI Biotyper Compass Explorer 4.1 from 20 individual spectra (1000 laser pulses for each) acquired from two different spots of duplicated samples. More than 10 spectra could not be measured from a single spot because of a depletion due to the relatively high laser energy used. Hierarchical trees were constructed from the MSPs in MALDI Biotyper Compass Explorer 4.1. This approach has been shown consistent as regards to the output with constructing dendrograms based on 16S rRNA sequencing data [10]. The dendrogram creation method used was a standard method offered by the software with a default set of parameters: the distance measure was set at correlation; the linkage was set at average. Reference proteomes for *Synechococcus* cyanobacteria were downloaded as a single FASTA formatted file from the UniProt database at https://www.uniprot.org/proteomes/ in July 2020. Theoretical molecular masses—monoisotopic and average—were then calculated based on the proteome sequences and the average masses used to assign the identified spectral peaks with a mass tolerance of ±2 Da. This calculation and assignment were performed by a program application pFilter available at https://software.cr-hana.upol.cz/pfilter/ (Raus, M. and Šebela, M., unpublished results). Differences in the representation of peaks towards the control were determined by means of the same in-house software tool using peaklists from the MSPs. All pairs of the sample and control spectra were also compared for differential peaks visually in mMass 5.5.0 using a flip mode to align the control in an upside-down orientation for clarity [59]. Possible annotations for yet uncharacterized proteins were analyzed using Conserved Domains Database search tool available at https://www.ncbi.nlm.nih.gov/Structure/cdd/wrpsb.cgi/ (accessed in December 2020).

## Data Availability

Research data are available by the authors upon a request.

## Supplementary Materials

The following are available online, Figure S1: Hierarchical trees of *Synechococcus* sp. UPOC S4 cells based on IC MALDI-TOF mass spectra, File S1: Assigning of peaks from intact cell MALDI-TOF mass spectra of *Synechococcus* sp. UPOC S4 to amino acid sequences from the UniProt database, File S2: TXT formatted spectral files.
